# Setting Up a Cultural Psychiatry Group (CPG) at Birmingham and Solihull Mental Health Foundation Trust (BSMHFT) – the Achievements, the Pitfalls and What We Have Learnt

**DOI:** 10.1192/bjo.2022.146

**Published:** 2022-06-20

**Authors:** Megan Parsons, Razan Saeed, Zulfiquar Fathima

**Affiliations:** Birmingham and Solihull Mental Health NHS Foundation Trust, Birmingham, United Kingdom

## Abstract

**Aims:**

Whilst psychiatry training is both demanding and enjoyable, we feel that the theory does not fully capture what we see in our everyday work. For many of our patients, it fails to contextualise their experience within their socio-politico-economic environment. Working with patients with different ways of seeing, knowing and being necessitates an awareness of one's own and the other's sociocultural world in order to build an empathetic and empowering doctor-patient partnership.

We started a CPG with the hope of exploring resources from those whose perspectives are often left out of our training experience, with a view towards integrating these voices together with our clinical experiences and training program. We aimed to create a space where we could regularly explore the experiences of ourselves, our patients, and the societies in which we work, reflecting on the conscious and unconscious roles we inhabit.

Our aims for the space were to: recognise that everyone will have something valuable to contribute. Cultivate a space where people feel able to share openly. Maintain the safety of the space through compassion and accountability. - Show willingness to be uncomfortable but continue engaging in order to learn together.

**Methods:**

In Spring 2021, four Core Psychiatry Trainees from BSMHFT met together to plan a trust-wide CPG. There were three clear cycles of CPG meetings, the first consisting of member led sessions, the second outside speaker led sessions and the third an amalgamation of the two. Meetings were continually reviewed throughout each cycle with more formal evaluation and alteration at the end.

**Results:**

The first part of the discussion focuses on what went well with the themes being:

- Developing habits of lifelong learning
- Developing relationships with peers and the community
- Creating space for self and group reflection
- Developing transferable skills (leadership, management, teamwork).

The second part of the discussion focusses on the problems that the group encountered and how they were overcome. The main themes being:

- Technology
- Communication
- Engagement
- Management.

**Conclusion:**

At an individual level, this experience has been challenging but rewarding and we have received overwhelmingly positive feedback. Locally, the BSMHFT CPG has been invited to work with our trust on their “inequality strategy”, as well as universities and organisations represented by outside speakers. Nationally, the blueprint laid out in our conclusion aims to help those wanting to set up a similar group in their area benefit from our experience.

